# The one-carbon metabolism as an underlying pathway for placental DNA methylation – a systematic review

**DOI:** 10.1080/15592294.2024.2318516

**Published:** 2024-03-14

**Authors:** Marjolein M van Vliet, Sam Schoenmakers, Joost Gribnau, Régine P.M. Steegers-Theunissen

**Affiliations:** a Department of Obstetrics and Gynaecology, Erasmus MC, Rotterdam, the Netherlands; b Department of Developmental Biology, Erasmus MC, Rotterdam, the Netherlands

**Keywords:** DoHAD, epigenetics, nutrition, human

## Abstract

Epigenetic modifications, including DNA methylation, are proposed mechanisms explaining the impact of parental exposures to foetal development and lifelong health. Micronutrients including folate, choline, and vitamin B_12_ provide methyl groups for the one-carbon metabolism and subsequent DNA methylation processes. Placental DNA methylation changes in response to one-carbon moieties hold potential targets to improve obstetrical care. We conducted a systematic review on the associations between one-carbon metabolism and human placental DNA methylation. We included 22 studies. Findings from clinical studies with minimal ErasmusAGE quality score 5/10 (*n* = 15) and *in vitro* studies (*n* = 3) are summarized for different one-carbon moieties. Next, results are discussed per study approach: (1) global DNA methylation (*n* = 9), (2) genome-wide analyses (*n* = 4), and (3) gene specific (*n* = 14). Generally, one-carbon moieties were not associated with global methylation, although conflicting outcomes were reported specifically for choline. Using genome-wide approaches, few differentially methylated sites associated with S-adenosylmethionine (SAM), S-adenosylhomocysteine (SAH), or dietary patterns. Most studies taking a gene-specific approach indicated site-specific relationships depending on studied moiety and genomic region, specifically in genes involved in growth and development including *LEP*, *NR3C1, CRH*, and *PlGF*; however, overlap between studies was low. Therefore, we recommend to further investigate the impact of an optimized one-carbon metabolism on DNA methylation and lifelong health.

## Introduction

Foetal exposures within the intra-uterine environment can affect health outcomes of the offspring during the life course. This is known as the developmental origins of health and disease (DOHaD) paradigm [1,2]. Epigenetic modifications can affect gene-expression and consequently cell function without changing the DNA sequence and are proposed underlying mechanisms connecting parental exposures to gamete maturation, embryonic and foetal development, and long-term health outcomes in the offspring [1,3].

The best characterized epigenetic mechanism is DNA methylation. DNA methylation is regulated by DNA methyltransferases (DNMTs), which transfer methyl groups predominantly to the C-5 position of a cytosine at a cytosine-phosphate-guanine (CpG) site [4,5]. Methylation of CpGs in regulatory genomic regions like promoters typically leads to gene silencing while non-genic, repetitive DNA sequences, such as transposable elements (e.g., LINE-1 and Alu elements), are often heavily methylated to maintain genomic stability and can serve as markers for global methylation [4–6].

During the periconception period, starting 14 weeks before conception till 10 weeks after conception, significant epigenetic modifications with potential effects along the life course take place [7]. Most DNA methylation marks present in parental gametes are removed during the first cell divisions in the zygote and blastocyst stages, followed by the establishment of *de novo* DNA methylation most prominent after implantation. *In utero*, proper *de novo* DNA methylation is essential for key biological processes involved in regulation of gene expression during development and differentiation [4–8]. Therefore, the periconception and pregnancy period is a vulnerable time span for epigenetic disruptions.

**Figure 1.**
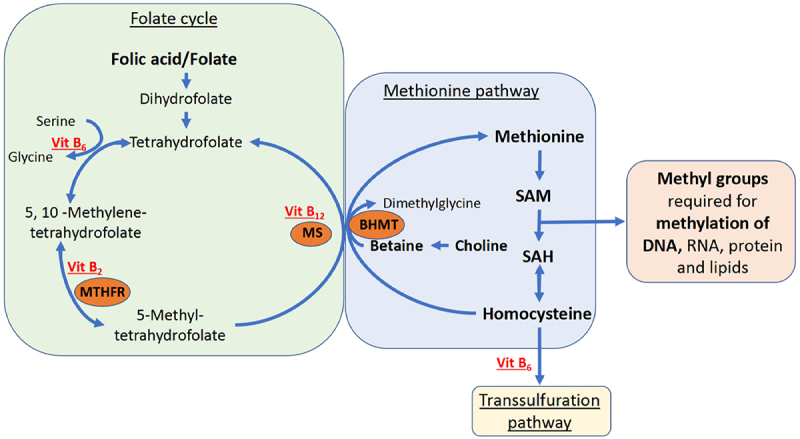
Simplified overview of the one-carbon metabolism.

The one-carbon metabolism provides methyl groups required amongst others for DNA methylation. Herein, methionine is converted to the major methyl donor s-adenosylmethionine (SAM), which in turn is converted to s-adenosylhomocysteine (SAH) thereby donating its methyl group. Other essential substrates of the one-carbon metabolism, here referred to as one-carbon moieties, include methyl donors such as folate and choline, and co-factors including vitamin B_2_, B_6_, and B_12_, that are all mostly derived from diet (Figure 1) [9].

Dietary methionine is converted to homocysteine via SAM and SAH. SAM is a major methyl donor for biological processes, including DNA methylation. The remethylation of homocysteine to methionine depends on other moieties including vitamin B12 as cofactor and 5 methyl tetrahydrofolate as substrate. MTHFR = methylenetetrahydrofolate reductase, BHMT = betaine-homocysteine S-methyltransferase, MS = methionine synthase, SAM = s-adenosylmethionine, SAH = s-adenosylhomocysteine

Evidence from predominantly animal studies indicates that differences in one-carbon metabolism during pregnancy can cause lasting DNA methylation changes in the offspring [7,10–17]. A famous example is that a methyl-supplemented diet fed to pregnant dams of the Agouti mouse increases DNA methylation of a regulating locus that results in a lower expression of the *Agouti* gene in their offspring, resulting in changed adiposity and coat colour [18,19]. In humans conceived during the Dutch Hunger Winter, an increased risk for obesity was observed and methylation differences in whole blood are found related to genes involved in growth and metabolism including lower methylation of insulin-like growth factor 2 (*IGF2*) gene [20]. Additionally, offspring of women who used periconceptional folic acid supplements had a higher methylation of *IGF2* in blood [15]. This illustrates that nutrition, particularly moieties of one-carbon metabolism, plays a crucial role in early life epigenetic programming with potential lifelong health consequences [7].

**Figure 2.**
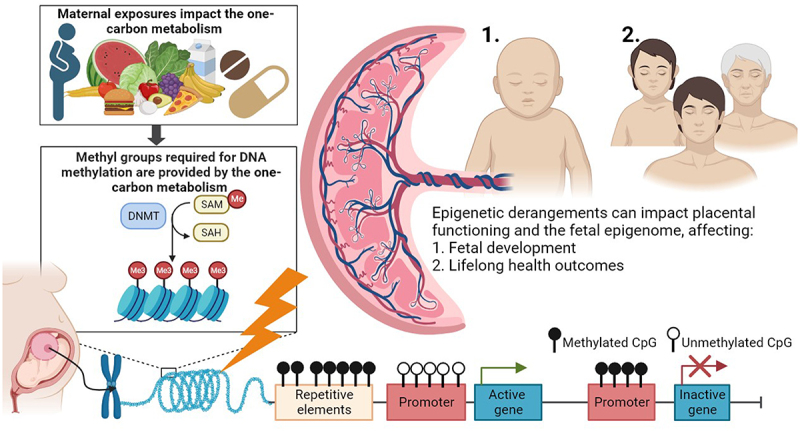
The one-carbon metabolism as an underlying pathway affecting placental DNA methylation and lifelong health outcomes of the offspring.

The placenta is an interesting target organ for DNA methylation studies investigating the relationship between maternal exposures including nutrition, one-carbon metabolism, and foetal programming as it forms the unique and indispensable interface between the mother and the developing embryo and foetus [21]. The changes observed in the placental DNA methylation are suggested to reflect alterations in placental functioning and as such impact foetal programming. The treatment of these adverse variations could provide targets for future preventative or therapeutic interventions (Figure 2) [22]. Additionally, we suggest that the presence of placental-originated cell free DNA (cfDNA) in maternal blood will become an opportunity to non-invasively assess placental DNA methylation profiles during early pregnancy, whereas the non-invasive collection of foetal tissues is not possible. This emphasizes the potential of the placenta as a proxy for foetal health conditions [23].

Maternal exposures such as nutrition and the use of vitamin supplements, are involved in the one-carbon metabolism, which provides methyl groups for DNA methylation processes (left). DNA methyltransferases (DNMTs) transfer methyl groups to the cytosine-phosphate-guanine sides (CpGs). Derangement of the one-carbon metabolism are proposed mechanisms affecting DNA methylation processes (bottom). The placenta is the intermediate between the maternal environment and embryonic and foetal growth and development. Epigenetic derangements can affect (1) directly the functioning of the placenta and subsequently foetal growth and development, and (2) the foetal epigenome with potential consequences for foetal growth and development, and 1 and 2 both have consequences for lifelong health outcomes in the offspring.

Previous reviews about the impact of the one-carbon metabolism in reproduction have mainly focussed on DNA methylation in tissues other than placenta including umbilical cord blood, focussed on animal studies and/or were limited to one specific one-carbon moiety [7,10–14,24–26]. Additionally, epigenome-wide association studies have specifically emerged over the last years [27]. Therefore, this systematic review aims to provide an overview of the current knowledge obtained from human studies investigating the impact of the one-carbon metabolism on placental DNA methylation. Epigenetic derangements in response to impaired one-carbon metabolism could hold potential targets for future therapeutic or preventative interventions, including prenatal diagnosis and obstetrical care.

## Methods

### Protocol and registration

The protocol for this systematic review was registered to the PROSPERO registry (PROSPERO 2023 CRD42023393358). The PRISMA Guidelines for systematic reviews and meta-analysis protocols were followed [28].

### Search strategy

Medline (on the Ovid platform), Embase, Web of Science Core Collection, Cochrane Central Register of Controlled Trials, and Google Scholar were searched until 12 July 2023. An expert research librarian was involved in setting up the search strategy. The search strategy included but was not limited to terms related to one-carbon metabolism like folate, homocysteine, and nutrition combined with placental related terms and key words related to epigenetics including DNA methylation. The full search is provided in Table S1.

### Study selection

Clinical studies were eligible if they studied one-carbon moieties during the preconception period and/or during pregnancy including birth and studied DNA methylation in human placental tissues. Since in practice, one-carbon moieties are consumed by foods and vitamin supplements, we also included articles investigating associations between dietary patterns reflecting the intake of one-carbon moieties or intake of multivitamin supplements. *In vitro* studies were eligible if they studied one-carbon moieties and DNA methylation in a human *in vitro* placental model. Review articles, conference abstracts, and articles not available in English language were excluded. MV and SS performed the title and abstract screening and the full-text review of remaining articles.

### Quality assessment of included studies

We used the ErasmusAGE quality score system for systematic reviews to assess the quality of included clinical studies [29]. Quality of included articles was assessed by MV and SS independently. All articles were scored based on five items and each item was scored zero, one or two points, leading to a score between 0–10 for each article. These items include study design (cross-sectional study = 0, longitudinal study = 1, intervention study = 2), study size (<50 patients = 0, 50–100 patients = 1, >100 patients = 2), method of measuring exposure and method of measuring outcome (both: no appropriate measure = 0, moderate quality of measure = 1, adequate measure = 2), and adjustments for confounders (no adjustment = 0, adjustment for key confounders = 1, adjustment for additional covariates or extra confounders = 2) (Table S2) [29]. We discussed all articles with an ErasmusAGE quality score of at least 5.

### Data extraction

Data extraction was done using a predefined form. Collected data included: study design, country of origin, year of publication, study population including sample size, method of measuring (exposure to) one-carbon moieties, timing of exposure (e.g., pre-and periconceptional, pregnancy), which part of placental tissue was studied (e.g., foetal or maternal side biopsy), focus of DNA methylation (global, genome-wide, or gene-specific approach), specified methylation technique. Lastly the main outcomes of the study related to DNA methylation were collected.

## Results

### Study selection

**Figure 3.**
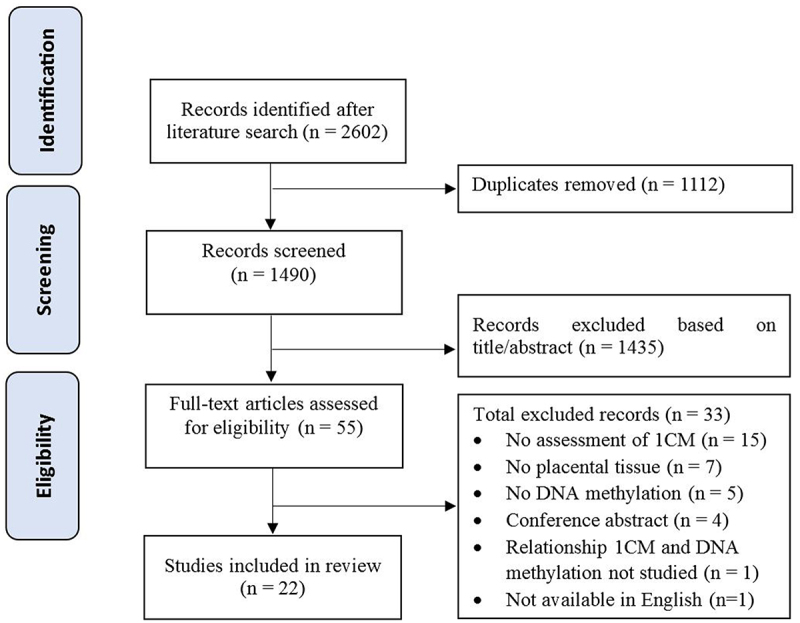
Flowchart of included and excluded articles. 1 CM = one-carbon moieties.

The literature search resulted in 1490 unique records after duplicate removal. After title and abstract screening, 1435 articles were excluded and a total of 55 full-text articles were assessed for eligibility of which 22 were included for the current review (Figure 3). The reasons for final exclusion included no exposure to or measurements of one-carbon moieties (*n* = 15), no placental tissue studied (*n* = 7), no DNA methylation as outcome (*n* = 5) and other (*n* = 6) (Figure 3).

### Study characteristics

The study characteristics and main outcomes of included studies are provided in Table 1. The included clinical studies were observational (*n* = 16) or clinical intervention studies (*n* = 3). Two solely *in vitro* studies were included [50,51] and one observational study combined clinical and *in vitro* work [43]. All included *in vitro* studies used human placental cell lines.

**Table 1.** Study characteristics and main findings of included studies ordered according to the quality score.

Author (year) – Country	Study design	Population and sample size	Exposure and timing	DNA methylation technique	Placental tissue	Main outcomes	Quality Score
Devi (2017) – India [30]	Randomized controlled trial	Healthy pregnant women with low serum vitamin B_12_ (<200 pmol/L) in first trimester (*n* = 66)	Group 1: 500 mL milk +10 mg vitamin B_12_/d (*n* = 23). Group 2: 500 mL milk + placebo (*n* = 20). Group 3: placebo (*n* = 23). Late first trimester till delivery.	Global methylation: LINE-1 methylation – MethyLight Gene specific: *VEGF* promoter — MS-HRM	Foetal side	Use of milk with or without vitamin B_12_ did not change *VEGF* promoter or LINE-1 methylation.	8
Dou (2022) – USA [31]	(Subgroup of) 2 prospective cohorts	Couples at increased risk for offspring with ASD. *N* = 70 (MARBLES cohort) + *N* = 88 (EARLI cohort)	Maternal multivitamin use in first month of pregnancy	Global methylation: Mean array methylation – MARBLES: 450K array EARLI: EPIC array Genome wide: individual CpGs – MARBLES: 450K array, EARLI: EPIC array	Foetal side (MARBLES) Central full thickness biopsy (EARLI)	Negative association between mean array methylation and vitamin use: −0.60% (CI −1.08, − 0.13; MARBLES); −0.52% (CI −1.04, 0.01; EARLI). No associations with individual CpGs after FDR correction. Using a less stringent threshold (of *p*<0.01), 9216 (MARBLES) and 3442 (EARLI) differentially methylated CpGs of which ±95% were hypomethylated in women using vitamins (average −4%). Identified CpGs were enriched in neuronal developmental pathways.	8
Lecorguille (2022) — France [32]	Prospective cohort	Healthy pregnant women (*n*=573)	1. Dietary intake in year before pregnancy reflecting differences in one-carbon moieties 2. Dietary supplements 3 months before and during pregnancy	Global methylation: LINE-1 and Alu repetitive elements methylation. —Pyrosequencing Genome wide: DMRs and individual CpGs —450K array	Foetal side, central	No associations between dietary patterns and individual CpGs or global methylation. ‘Varied and balanced’ diet associated with a DMR related to *NPDC1* (*p*=0.02). ‘Vegetarian tendency’ associated negatively with 2 DMRs related to *DLL1* (*p*=5.3.10^−7^, *p*=7.0.10^−9^) and *FAR1* (*p*=3.7.10^−9^, *p*=7.8.10^−3^). ‘Bread and starchy food’ associated negatively with *AS3MT* (*p*=0.02) and *GSDMD* (*p*=0.004) and positively with *SLC25A46* (*p*=0.01) and *ZNF175* (*p* =0.006) DMRs. Vitamin use associated with Alu repetitive elements methylation (β=0.40, *p*=0.005) compared to no vitamins. No associations for vitamin use only before *or* during pregnancy or with LINE-1 methylation.	7
Castillo-Castrejon (2021) — Guatemale & Pakistan [33]	Randomized controlled trial	Preconceptional women. Q1 and Q4 based on newborns’ birth length (*n*=24 in both Guatemale and Pakistan, *n*=48)	Group 1: Daily SQLNS ≥3 months before conception until delivery Group 2: No supplement use.	Gene specific: P2 promoter region of *IGF1* —Pyrosequencing	Four representative biopsies	SQLNS use did not change *IGF1* promoter methylation.	6
Jiang (2012) – USA [34]	Randomized controlled trial	Healthy women in third trimester (*n*=24)	Group 1: 480mg choline/d (*n*=12). Group 2: 930mg choline/d (*n*=12). 12 weeks trial, supplements till delivery	Global DNA methylation —LC-MS/MS Gene specific: *CRH*, *GNAS-AS1*, *LEP*, *IL10* promoters; the 5= untranslated exon 1F (and flanking regions) of *NR3C1*, DMR0 of *IGF2* —Base-specific cleavage and MS	Full-thickness biopsies from centre of each quadrant	Higher choline intake (930mg/d) increased global DNA methylation (4.4 ±0.2% VS 3.6 ± 0.2%, *p*=0.02). Higher choline intake (930mg/d) increased methylation of *CRH* (*p*=0.05) and *NR3C1* (*p*=0.002). Higher methylation was found for 4/15 *NR3C1* CpGs and no individual *CRH* CpG reached significance. Higher choline intake (930mg/d) decreased methylation of 1/11 *GNAS-AS1* CpG (*p*=0.02), average *GNAS-AS1* methylation was not changed. Higher choline intake did not change methylation of DMR0 of *IGF2* or promoter regions of *IL10* or *LEP*.	6
Liu (2019) — China [35]	Case-controls from prospective cohort	34 SGA cases, 62 AGA controls	Preconceptional folic acid supplement use	Gene specific: 2 *ANKRD20B* CpGs, 9 *FAM198A* CpGs; both were top ranked variable positions between 3 SGA and 3 AGA (450K array) – Pyrosequencing	Foetal side, central	In male offspring, lower methylation of 5/9 sites within *FAM198A* associated with preconceptional folic acid supplement use (*p*=0.047, *p*=0.050, *p*=0.039, *p*=0.026, *p*=0.043). No associations in female offspring or for *ANKRD20B*.	6
Loke (2013) – Australia [36]	(Subgroup of) prospective twin cohort	Monozygotic (*n*=34) and dizygotic (*n*=33) twin pairs	1. Folic acid supplements <12 weeks of gestation 2. Serum vitamin B_12_ + homocysteine at 28 weeks of gestation	Gene specific: 4 *IGF2/H19* DMRs —EpiTYPER MassARRAY	Unclear	Folic acid supplement use associated with lower methylation (−4%) of *H19* promoter DMR. No associations between folic acid supplement use and methylation of *IGF2/H19* ICR, DMR0 and DMR2 of *IGF2.* No associations between vitamin B_12_ or homocysteine and methylation of 4 *IGF2/H19* DMRs.	6
Nakanishi (2021) – Japan [37]	Prospective cohort	Healthy pregnant women (*n*=124)	Maternal plasma choline levels in first trimester, third trimester and at term, choline levels in cord blood.	Global methylation: LINE-1 methylation – Pyrosequencing Gene specific: CpGs sites in *MEST*, *MEG*, *H19*, *PPARA*, *PPARG*, *ADIPOQ*, *LEP*, *NADH*, *NDUFb6*, *NR3C1*, *RXRA*, *HSD11β2* —Pyrosequencing	Full-thickness biopsy, central	Negative associations between maternal choline and *PPARG1A*, *NR3C1*, *HSD11β2*, *PPARA* and *RXRA* methylation at all three time points (range: *r*=−0.188 — *r*=−0.452) and between cord blood choline and *NR3C1* (*r*=−0.20, *p*=0.033) and *PPARA* (*r*=0.223, *p*=0.017) methylation. Maternal choline in 1^st^ trimester associated negatively with *AdipoQ* methylation (*r*=−0.307, *p*=0.001) and 3^th^ trimester choline associated negatively with *AdipoQ* (*r*=−0.201, *p*=0.030) and *NDUFB6* (*r*=−0.214, *p*=0.021) methylation. No associations with *MEG3*, *H19*, *MEST* and *LEP*. LINE-1 methylation associated negatively with choline in 1^st^ and 3^th^ trimester (*r*= −0.205, *p*=0.022; *r*=−0.207, *p*=0.026) and in cord blood (*r*=−0.248, *p*=0.008).	6
Schmidt (2016) – USA [38]	Case-controls from prospective cohort	Couples at increased risk for offspring with ASD. ASD (*n*=24) and controls (*n*=23).	Food folate intake during pregnancy and vitamin use in first month of pregnancy	Global methylation: predefined partially- or highly methylated domains —MethylC-seq	Foetal side, midway cord insertion and periphery	No associations between folate intake (after FDR correction) or vitamin use and methylation of predefined partially- or highly methylated domains.	6
Heil (2019) – The Netherlands [39]	Case-control study	Pregnancies with PE (early onset *n*=11, late onset *n*=11), FGR (*n*=20), PTB (*n*=15) and controls (*n*=25)	SAM and SAH concentrations in placental tissue	Gene specific: 1 CpG in the 5’flanking region of the *PIGF* gene —EpiTYPER MassARRAY	Foetal side, 4 biopsies 3cm around cord insertion, removal 2mm of top layer.	SAM and SAM:SAH ratio were positively associated with *PIGF* methylation (*r*=0.31, *p*=0.006; *r*=0.24, *p*=0.04, respectively).	5
Khot (2017) – India [40]	Case-control study	Term (*n*=69) and preterm (*n*=71) deliveries in healthy women	Plasma vitamin B_12_, folate and homocysteine just after delivery	Gene specific: *MTR* promoter — Pyrosequencing	Random biopsies from decidual side	Vitamin B_12_ associated negatively with methylation of 3/7 investigated CpGs in the *MTR* promoter (*r* =-0.208, *p*=0.049; *r* =-0.248, *p*=0.017; *r*=−0.298, *p*=0.004). Women delivering preterm had lower plasma folate and higher homocysteine compared to term controls. Mean *MTR* promoter methylation was 0.5% higher (*p*< 0.01) in preterm births. 5/7 studied CpGs had higher methylation in preterm placentas only after vaginal deliveries. No associations between homocysteine or folate and DNA methylation were investigated.	5
Moeini (2022) – Iran [41]	Case-control study	Term (*n*=25) and preterm (*n*=25) deliveries in healthy women	Serum folate and vitamin B_12_, timing unclear	Gene specific: *MMP-9* promoter —Epitect Methyl-II PCR assay kit	Unclear	Folate and vitamin B_12_ associated with *MMP-9* promoter methylation (*r*=0.58, *p*<0.001; *r*=0.68, *p* <0.001, respectively).	5
van Otterdijk (2023) – USA [42]	Subgroup of cohort study	Mother-infants dyads homozygous for the C or T allele of MTHFR C677T polymorphism (both *n*=45)	Red blood cell folate, vitamin B_12_, SAM, SAH in cord blood.	Global methylation: Average methylation of the following regions: CpG islands, shores, shelves and open sea —450K array Genome wide: individual CpGs —450K array	Foetal side, near umbilical cord	RBC folate correlated positively with methylation of CpG islands and ‘shelves’ for the total study population and in ‘open sea’ and ‘shores’ only for the TT genotype. No significant associations between individual CpGs and RBC folate after multiple testing correction. SAM:SAH ratio correlated positively with methylation of ‘open sea’ and ‘shore’ regions and with individual loci located in *RAD54L2*, *ETS2*, *ZNF836* and *ABAT*. No significant associations between SAM or SAH and region-specific DNA methylation patterns. SAM significantly associated with 2 loci located in *LOXL3* and *GNL1* gene bodies and SAH with 1 CpG in *ZC3H12D* CpG island. No associations between vitamin B_12_ and regional methylation or methylation of individual loci.	5
Rahat (2022)* — India [43]	Case-control study	Uncomplicated terminations in 1^st^/2^nd^ trimester (both *n*=30), term and PE cases (both *n*=30)	Folate levels in placental villi	Global methylation: —ELISA-based kit and LINE-1 methylation —MS-HRM	Placenta villi	No significant associations between folate levels in placental villi and global methylation.	5
Sletner (2021) – Norway [44]	(Subgroup of) prospective cohort	Healthy pregnant women, ethnic south Asian (*N*=40) or European (*n*=40)	Serum folate and vitamin B_12_ at enrolment (<20 weeks of gestation)	Gene specific: 13 CpGs in the Transcription start site of *LEP* — Pyrosequencing	Full thickness biopsy, central	Folate associated with methylation of 9/13 CpGs in a multivariate model (range: β=0.16—0.26, *p*=0.003—p=0.03) but not in univariate models. No associations between vitamin B_12_ and methylation of 13 *LEP* CpGs in univariate analyses, negative associations with 2/13 CpGs in multivariate models (β=-0.8, *p*=0.02; β=-0.02, *p*=0.02).	5
Ge (2015) — China [45]	Case-control study	Pregnancies with PE (*n*=127) and controls (*n*=132)	Homocysteine in maternal plasma on admission	Gene specific: *MTHFR* promoter — Methylation specific PCR	Central biopsy and 1 from either side of it	*MTHFR* methylation indices were higher in PE (26.8% VS 15.2%, *p*<0.05) and homocysteine was higher in PE (*p*<0.05). No association between homocysteine and methylation was investigated.	4
Pinunuri (2020) – Chile [46]	Case-controls from prospective cohort	Term (*n*=16) and preterm (gestational age 32–36 weeks, *n*=23) deliveries in healthy women	Folate and vitamin B_12_ in umbilical cord blood	Gene specific: CpG island upstream of transcription initiation site *FOLR1* —MS-HRM	Chorionic and basal plates biopsies near cord insertion	Cord blood folate associated with *FOLR1* methylation in the chorionic plate (Rho =0.665, *p*≤0.05) but not in the basal plate. There were no associations between cord blood vitamin B_12_ and *FOLR1* methylation.	4
Tserga (2017) – USA [47]	(Subgroup of) prospective cohort	Healthy Caucasian women homozygous for C or T allele of MTHFR C677T polymorphism in mother and child (*n*=48)	1. Folic acid supplements started before VS during pregnancy 2. Red blood cell folate, SAM, SAH, vitamin B_12_ in cord blood	Gene specific: *H19ICR* —Pyrosequencing	Foetal side near cord insertion	No associations between preconceptional start of FA, red blood cell folate, vitamin B_12_ or SAM levels in cord blood and *H19ICR* methylation. *H19ICR* methylation associated with SAM:SAH ratio (*R*=0.38) and negatively with log(SAH) (*R*=−0.40) in cord blood.	4
Zhu (2019) – USA [48]	(Subgroup of) prospective cohort	Couples at increased risk for offspring with ASD. ASD cases (*n*=20) and controls (*n*=21)	Vitamin use in first month of pregnancy	Gene specific: *CYP2E1* and *IRS2 DMRs (2* ASD-associated DMRs) — Pyrosequencing Genome wide: DMRs —WGBS	Foetal side	Vitamin use associated with 376 DMRs. Genes were enriched for neuron fate commitment, central nervous system development, regulation of transcription and of phosphatidylinositol 3-kinase activity functions. Vitamin use associated negatively with *IRS2* DMR methylation (*p*=0.039) and positively but not significantly with *CYP2E1* (*p*=0.118).	4
Kulkarni (2011) – India [49]	Case-control study	Pregnancies with term PE (*n*=30), preterm PE (*n*=27) and controls (*n*=30)	Vitamin B_12_ in maternal plasma at delivery, homocysteine in maternal plasma in subset (16 controls, 28 term PE, 19 preterm PE)	Global DNA methylation —Methylamp^TM^ Global DNA Methylation Quantification Kit	5 different villi biopsies	Vitamin B_12_ was higher in preterm PE (261 ng/mL) and term PE (191 ng/mL) compared to controls (145 ng/mL) and homocysteine was also higher in preterm PE (17.59 uM) and term PE (14.43 uM) compared to controls (11.01 uM). Mean global methylation was higher in term PE (0.68%) and preterm PE (0.72%) compared to controls (0.53%), *p* <0.05. No association between vitamin B_12_ or homocysteine and DNA methylation was studied.	3
*In vitro studies using human placental cell lines*							
Jiang (2016) – USA [50]	*In vitro* study	BeWo cell line	Incubation for 96 hours with 1mM choline chloride	Global methylation —ELISA-based kit	NA	Choline did not change global DNA methylation.	NA
Rahat (2017) — India [51]	*In vitro* study	JEG-3 and HTR-8/SVneo cell lines, first trimester cytotrophoblasts	Incubation for 48 hours with folic acid (10^−7^ M and 10^−4^ M)	Gene specific: DMRs of *SNRPN*, *PEG10* and *MEST* — MS-HRM	NA	Folic acid decreased *SNRPN* methylation in JEG-3, HTR-8/SVneo and in cytotrophoblasts. At 10^−7^ M, methylation decreased respectively by 1.5 (*p*<0.01), 1.2 (*p*<0.05) and 1.2 (*p*<0.05) and at 10^−4^ by 1.8 (*p*<0.001), 2.2 (*p*<0.001) and 1.4 (*p*<0.01) fold. Folic acid did not change *PEG10* or *MEST* methylation.	NA
Rahat (2022)* — India [43]	*In vitro* study	JEG-3 and HTR-8/SVneo cell lines	Incubation for 48 hours with folic acid (10^−7^ M and 10^−4^ M)	Global methylation: —ELISA-based kit and LINE-1 methylation —MS-HRM Gene specific: *RASSF1A*, *P16*, *RB1*, *PRKCDBP*, *APC*, *c-myc*, *c-jun*, *VEGF*, *EGFR*, *hTERT*, *MMP2*, *MMP-9*, *TIMP1*, *TIMP2* —MS-HRM	NA	Folic acid incubation did not change LINE-1 methylation. Global methylation decreased in dose-dependent manner. JEG-3 cells: − 3.7% and −4.9% at 10^−7^ M and 10^−4^ M. HTR-8/SVneo cells: − 4.3% and −5.7% at 10^−7^ M and 10^−4^ M (*p*<0.001). In JEG-3 cells, folic acid increased methylation of *APC* (1.14x at 10^−7^ M; 1.28x at 10^−4^ M) and *P16* (5x at 10^−7^ M) and decreased for *c-jun* (1.1x at 10^−7^ M). In HTR-8/SVneo cells, methylation increased for *APC* (4.1x at 10^−7^ M) and decreased for *P16 (*2.4x at 10^−7^ M and 3.5x at 10^−4^ M), *c-myc* (1.8x at 10^−7^ M and 2.5x at 10^−4^ M) and *c-jun* (2.14 x at 10^−7^ M and 5.5x at 10^−4^ M)	NA

*Same study.

WGBS = Whole-Genome Bisulfite Sequencing, LINE-1 = Long Interspersed Nuclear Element-1, ASD = autism spectrum disorder, SGA = small for gestational age, AGA = appropriate growth for gestational age, SQLNS = small quantity lipid-based micronutrient, CpG = cytosine-phosphate-Guanine, DMR = Differentially Methylated Region, SAM = S-adenosylmethionine, SAH = S-adenosylhomocysteine, PE = preeclampsia, FGR = foetal growth restriction, PTB = preterm birth, NA = not applicable, LC-MS/MS = Liquid Chromatography with tandem mass spectrometry, MS-HRM = Methylation-sensitive high-resolution melt analysis, MS = mass spectrometry, PCR = polymerase chain reaction.

One-carbon moieties that were studied in the clinical studies were folate/folic acid, (*n*=9), vitamin B_12_ (*n*=9), homocysteine (*n*=4), choline (*n*=2), SAM and SAH (*n*=3), combination supplements (*n*=4), and dietary patterns reflecting differences in intake of one-carbon moieties (*n*=1). Two *in vitro* studies investigated the effect of folate and one studied the effect of choline on DNA methylation (Table 1). Results are discussed below for the different included moieties.

Most clinical studies used a gene-specific approach (*n*=14/20). Four used a genome-wide approach and global DNA methylation was investigated in nine studies. Some studies used multiple approaches in the same cohort. Table 2 summarizes the studies that investigated the associations for all three approaches in clinical studies with an ErasmusAGE quality score of at least 5 points. Included clinical studies scored between 3 and 8 points (median 5) (Table S2).

**Table 2.** Direct associations between one-carbon moieties and global methylation, genome-wide methylation or gene-specific methylation in included clinical studies with an ErasmusAGE quality score of at least 5 out of 10 points.

Outcome	Exposure	Methylation	Ref
Global methylation	Dietary folate	=	[38]
	Folate in placental villi	=	[43]
	Umbilical cord RBC folate	↑/=	[42]
	Vitamin B_12_ supplements	=	[30]
	Umbilical cord vitamin B_12_	=	[42]
	Choline supplements	↑	[34]
	Choline in maternal blood: first trimester/third trimester/term	↓ / ↓ / =	[37]
	Choline in cord blood	↓	[37]
	Umbilical cord SAM SAH SAM:SAH Dietary patterns: ↑B-vitamins, choline, methionine ↓betaine ↑B_6_, folate, betaine ↓ B_12_ ↑Betaine ↓B_2_, B_6_, folate	= = ↑/= = = =	[42][32]
	Vitamin use in first month of pregnancy	=	[38]
		↓/ =	[31]
	Vitamin use before and during pregnancy Vitamin use before or during pregnancy	↑ / = = / =	[32][32]
Genome-wide	**Exposure**	**Genes linked to identified CpGs**	
	Umbilical cord RBC Folate	**=**	[42][32]
	Umbilical cord vitamin B_12_	**=**	
	Umbilical cord SAM SAH SAM:SAH Dietary patterns ↑B-vitamins, choline, methionine ↓betaine ↑B_6_, folate, betaine ↓ B_12_ ↑Betaine ↓B_2_, B_6_, folate	LOXL3 GNL1 *ZC3H12D* *RAD54L2 ETS2 ZNF836 ABAT* **Genes linked to identified DMRs** *NPDC1* *DLL1 FAR1* *AS3MT GSDMD SLC25A46 ZNF175*	
	Dietary patterns Vitamin use in first month of pregnancy	Individual CpGs = Individual CpGs = *	[31]
Gene-specific	**Exposure**	**Gene of interest**	
	Start folic acid preconceptional compared to start during pregnancy	*FAM198A* ↓(boys)/= (girls) *ANKRD20B* =	[35]
	Folic acid use in first trimester	*H19* promoter DMR↓ 2 *IGF2* DMRs *IGF2/H19ICR =*	[36]
	Maternal serum folate	*MMP-9* ↑	[41]
		*LEP =*/↑	[44]
	B_12_ supplements	*VEGF =*	[30]
	B_12_ in maternal blood	*MMP-9* ↑	[41]
		*MTR* ↓	[40]
		4 *IGF2/H19* DMRs =	[36]
	Homocysteine in maternal blood	4 *IGF2/H19* DMRs =	[36]
	Choline supplements	*CRH NR3C1* ↑ *GNAS-AS1* ↓/= *IGF2 IL-10 LEP* =	[34]
	Choline in first trimester maternal blood	*AdipoQ PPARG1A NR3C1, HSD11β2 PPARA RXRA* ↓ *MEG3 H19 MEST LEP NDUFB6* =	[37]
	Choline in third trimester maternal blood	*AdipoQ PPARG1A NR3C1, HSD11β2 PPARA NDUFB6 RXRA* ↓ *MEG3 H19 MEST LEP* =	[37]
	Choline in term maternal blood	*PPARG1A NR3C1 HSD11β2, PPARA RXRA* ↓ *AdipoQ MEG3 H19 MEST LEP NDUFB6* =	[37]
	Choline in cord blood	*NR3C1 PPARA* ↓ *AdipoQ PPARG1A HSD11β2 RXRA MEG3 H19 MEST LEP NDUFB6* =	[37]
	Placenta SAM and SAM:SAH	*PIGF* ↑	[39]
	Small quantity lipid-based micronutrient use	*IGF1* =	[33]

* No associations with individual CpGs after FDR correction. Using a less stringent threshold of p<0.01, 9216 (MARBLES) and 3442 (EARLI) differentially methylated CpGs of which ±95% was hypomethylated in vitamin groups (average -4%) were identified. Identified CpGs were enriched in neuronal developmental pathways.

Abbreviations: DMR = differentially methylated region, CpG = Cytosine-phosphate-Guanine, RBC = Red Blood Cell, SAM = S-adenosylmethionine, SAH = S-adenosylhomocysteine, FDR = False Discovery Rate.

### Metabolic determinants of one-carbon metabolism and placental DNA methylation outcomes

#### Folate/Folic acid

##### Dietary intake/supplement use

One study (ErasmusAGE score 6) assessed dietary folate intake during pregnancy by Food Frequency Questionnaires (FFQ) and found no association with methylation of partially- or highly methylated domains after controlling for the False Discovery Rate (FDR) [38].

*Liu et al*. (ErasmusAGE score 7) showed lower methylation of 5 out of 9 investigated sites within *FAM198A* in women who started using folic acid supplements before pregnancy in male offspring (p-values between 0.026 — 0.050) but not in female offspring, indicating a sex-specific impact on DNA methylation. No associations were found between use of folic acid supplements and methylation of 2 *ANKRD20B* sites [35]. *FAM198A* and *ANKRD20B* sites were investigated, after these were formerly identified as top-ranked differentially methylated positions between 3 small for gestational age (SGA) children and 3 appropriate growth for gestational age (AGA) controls. *Loke et al*. (ErasmusAGE score 6) showed lower methylation of the differentially methylated region (DMR) of the *H19* promoter in women using folic acid supplements in first trimester compared to no use of folic acid supplement, but found no methylation differences in the *IGF2/H19* imprinted control region (ICR) or two *IGF2* DMRs [36].

##### Blood.

###### Maternal

In a multivariate model, folate levels associated positively with methylation of 9 out of 13 investigated CpGs at the transcription start site of *LEP* (range: β = 0.16—0.26, *p *= 0.003—p = 0.03) but not in univariate analyses (ErasmusAGE score 5) [44]. In another study, folate was positively associated with methylation of the *MMP-9* promoter (*r *= 0.58, *p *< 0.001) [41] (both ErasmusAGE score 5).

###### Foetal (umbilical cord)

*Van Otterdijk et al*. (ErasmusAGE score 5) investigated average methylation of CpG islands, defined as genomic regions between 200 and 500 base pairs with >50% CG content, the 0–2 kb and 2-4kb up- and downstream regions next to CpG-islands, referred to as ‘shores’ and ‘shelves’ respectively, and CpGs outside these regions referred to as ‘open sea.’ Additionally, they took a genome-wide approach investigating single CpGs. The mother-infant dyads in their study were either homozygous for the wild-type MTHFR677 genotype or for the MTHFR C677T variant. The MTHFR C667T polymorphism leads to reduced methylenetetrahydrofolate reductase (MTHFR) activity which is involved in the one-carbon metabolism, leading to higher homocysteine and lower SAM concentrations (Figure 1). In the total study population, red blood cell (RBC) folate was positively associated with methylation of ‘shelve’ regions and CpG island. In mother-infant dyads homozygous for the MTHFR C677T variant, a positive association was also found between RBC folate and methylation of ‘open sea’ and ‘shore’ regions. No associations between individual CpGs and RBC folate remained significant after correcting for multiple testing [42].

##### Other

*Rahat et al. (2022)* (ErasmusAGE score 5) found no association between folate levels within placental villi and global DNA methylation or LINE-1 methylation [43].

##### *In vitro* studies

Investigation of DNA methylation in JEG-3, a placental choricocarcinoma-derived cell line and in HTR-8/SVneo cell line, a model of extra-villous trophoblast, in response to folic acid treatment showed no changes in LINE-1 methylation [43]. In contrast, global methylation decreased in both cell lines in a dose-dependent manner with the largest effect in HTR-8/SVneo cells, where a 4.3% decrease (*p *< 0.001) was found at 10^−7^ M folic acid, comparable to the physiological range of 400–600 µg/day, and a 5.7% decrease (*p*<0.001) was found at a much higher concentration of 10^−4^ M folic acid. Taking a gene-specific approach, promoter methylation of the tumour suppressor gene *APC* increased in both cell lines in response to folic acid, while for *P16* an increase was observed in JEG-3 cells while methylation decreased in HTR-8/SVneo cells. Methylation of the promoter regions of oncogenes *c-myc* and *c-jun* decreased in both cell lines [43]. No changes were found in the *MMP-9* promoter region. In a comparable *in vitro* study, methylation in the promoter regions of *SNRPN*, *PEG10*, and *MEST* was studied [51]. Folic acid suppletion decreased methylation of *SNRPN* in a dose-dependent manner in JEG-3, HTR-8/SVneo cell lines and in cytotrophoblasts. No changes were found for *PEG10* and *MEST* [51].

#### Interim conclusions – Folate/folic acid

Mixed results have been reported for the relationship between folate/folic acid and markers of global placental DNA methylation. Only one study took a genome-wide approach and identified no significant associations between methylation of single CpGs and cord blood folate. When taking a gene-specific approach, several – but not all – investigated sites associated with folate/folic acid (Table 2). This indicates site-specific relationships between DNA methylation and folate/folic acid.

#### Vitamin B_12_

##### Supplement use

In a randomized trial in Indian women with low vitamin B_12_ levels, LINE-1 methylation and *VEGF* promoter methylation did not differ between women who used vitamin B_12_ supplements from end first trimester till delivery as compared to women without vitamin B_12_ supplements (ErasmusAGE score 8) [30].

##### Blood

###### Maternal

No associations were found between vitamin B_12_ levels at 28 weeks of gestation and 4 investigated *IGF2/H19* DMRs (ErasmusAGE score 6) [36]. Contrary, vitamin B_12_ levels in first half of pregnancy associated with lower methylation of 2 out of 13 investigated CpGs at the transcription start site of *LEP* (β = −0.8, *p *= 0.02; β = −0.02, *p *= 0.02) [44] and vitamin B_12_ associated with *MMP-9* promoter methylation (*r *= 0.68, *p *< 0.001) (both ErasmusAGE score 5) [41]. A negative association was found between plasma vitamin B_12_ at time of delivery and methylation of 3 out of 7 investigated CpGs in the *MTR* promoter region (*r *= −0.208, *p *= 0.049; *r *= −0.248, *p *= 0.017; *r *= −0.298, *p *= 0.004) (ErasmusAGE score 5) [40].

###### Foetal (umbilical cord)

*Van Otterdijk et al*. found no significant associations between vitamin B_12_ and methylation of CpG islands, ‘shores,’ ‘shelves,’ or ‘open sea’ regions or with methylation of individual CpGs when taking a genome-wide approach (ErasmusAGE score 5) [42].

#### Interim conclusions – Vitamin B_12_

Vitamin B_12_ did not associate with different markers of global placental DNA methylation or with methylation of individual CpGs taking a genome-wide approach. In a gene-specific approach, several investigated sites were associated with vitamin B_12_, underlining again the site-specific relationship between DNA methylation and vitamin B_12_ (Table 2).

#### Homocysteine

##### Blood

###### Maternal

No associations were found between homocysteine and 4 studied *IGF2/H19* DMRs (ErasmusAGE score 6) [36].

##### Choline

###### Supplement use

*Jiang et al*. randomized women in third trimester to either 480mg or 930mg choline per day for 12 weeks (ErasmusAGE score 6). They studied global DNA methylation and methylation of regions related to cortisol-regulating genes *CRH* and *NR3C1*, methylation of DMR0 of *IGF2* and methylation of *LEP*, *GNAS-AS1*, and *IL10* promoter regions. Women in the 930mg group had higher global methylation compared to the 480mg group (4.4 ±0.2% VS 3.6 ±0.2%, *p *= 0.02) and higher methylation of the *CRH* promoter region (*p*=0.05) and the 5= untranslated exon 1F (and flanking regions) of *NR3C1* (*p*=0.002). Of the individual CpGs, 4 out of 15 studied *NR3C1* CpGs showed higher methylation while none of the 5 studied individual CpGs for the *CRH* region reached significance. Although 1 out of 11 studied *GNAS-AS1* CpGs was lower methylated in the 930mg group (*p *= 0.02), average *GNAS-AS1* methylation did not differ between groups. No differences were found in methylation of *IGF2*, *IL10*, or *LEP* promoter regions [34].

##### Blood

###### Maternal

One study determined maternal plasma choline in first trimester, third trimester, and at term, and investigated methylation levels of 12 genes involved in foetal growth, lipid and energy metabolism, or adipogenesis and DNA methylation of LINE-1 elements (ErasmusAGE score 6). A negative modest association was found between DNA methylation of *PPARG1A*, *NR3C1*, *HSD11β2*, *PPARA*, and *RXRA* and maternal choline at all three time points (range: *r *= −0.188 — *r *= −0.452). For *AdipoQ* a negative association with maternal choline levels was observed in first and third trimester (*r *= −0.307, *p *= 0.001; *r *= −0.201, *p *= 0.030) and for *NDUFB6* only in third trimester (*r *= −0.214, *p *= 0.021). No associations were found with *MEG3*, *H19*, *MEST*, and *LEP* methylation. LINE-1 methylation was negatively associated with maternal choline in first and third trimester (*r *= −0.205, *p *= 0.022; *r *= −0.207, *p *= 0.026) [37].

###### Foetal (umbilical cord)

The same study showed a negative association between choline in cord blood and methylation of *NR3C1*, *PPARA*, and LINE-1 (*r*=−0.20, *p*=0.033; *r*=−0.223, *p*=0.017; *r*=−0.248, *p*=0.008). No associations were found with methylation of *MEG3*, *H19*, *MEST*, *LEP*, *AdipoQ*, *NDUFB6*, *PPARG1A*, *HSD11β2*, or *RXRA* [37].

###### *In vitro* studies

In an *in vitro* study using BeWo cells as a model for first trimester trophoblast, global DNA methylation was not altered by choline treatment [50].

#### Interim conclusions – Choline

Mixed results have been reported for choline and markers of global placental DNA methylation as well as for choline and *NR3C1* methylation. Several other relationships between DNA methylation of specific genes and choline intake or blood levels were found depending on studied site and moment of measuring choline, indicating site-specific relationships between DNA methylation and choline which may differ depending on timing of exposure (Table 2).

#### S-adenosylmethionine (SAM) and S-adenosylhomocysteine (SAH)

##### Blood

###### Foetal (umbilical cord)

The SAM:SAH ratio was positively associated with methylation of ‘open sea’ and ‘shore’ regions only in mother-infant dyads homozygous for the MTHFR C677T genotype. There were no significant associations between SAM:SAH and CpG island or ‘shelve’ regions and the observed positive associations with SAM and negative associations with SAH did not reach significance for the different genomic regions. When investigating individual CpGs genome-wide, limited associations were found. The SAM:SAH ratio associated with individual CpGs located in *RAD54L2*, *ETS2*, *ZNF836*, and *ABAT*, SAM associated with two loci located in *LOXL3* and *GNL1* gene bodies and SAH associated with methylation of one CpG in a *ZC3H12D* CpG island. Several other identified associations did not reach significance after correcting for multiple testing (ErasmusAGE score 5) [42].

###### Other measures

*Heil et al*. (ErasmusAGE score 5) studied SAM and SAH levels in placentas and methylation of LINE-1 and 1 CpG in the 5’ flanking region of *PlGF*. SAM and SAM:SAH ratio were positively associated with *PlGF* methylation (*r*=0.31, *p*=0.006; *r*=0.24, *p*=0.04, respectively). LINE-1 methylation associated with *PlGF* methylation (*r*=0.40, *p*<0.001) but no association between LINE-1 and SAM or SAH was studied [39].

#### Interim conclusions – SAM/SAH

A higher SAM:SAH ratio indicates higher availability of methyl group donors (Figure 1). Although only two studies investigated SAM and SAH, the SAM:SAH ratio in umbilical cord blood associated positively with markers of global methylation depending on genotype, and SAM, SAH, and SAM:SAH associated with methylation of a few individual CpGs in a genome-wide study. Both placental SAM and SAM:SAH ratio associated positively with *PlGF* methylation (Table 2). This suggests that SAM and SAH can impact placental DNA methylation both at a regional/global level as well as at gene-specific sites.

#### Combinations

##### Dietary patterns

*Lecorguille et al*. (ErasmusAGE score 7) assessed dietary intake in the year before pregnancy and distinguished three dietary patterns [32]. After adjustment for vitamin use, the ‘varied and balanced’ diet which is rich in B vitamins, choline, and methionine but low in betaine showed a positive association with a DMR related to *NPDC1* (*p*=0.02). A ‘vegetarian tendency’ diet which is rich in vitamin B_6_, folate, and betaine but low in vitamin B_12_ showed a significant negative association with 2 DMRs related to *DLL1* (*p *= 5.3.10^−7^, *p *= 7.0.10^−9^) and *FAR1* (*p *= 3.7.10^−9^, *p *= 7.8.10^−3^). Lastly, ‘bread and starchy food’ diet which is rich in betaine but low in vitamin B_2_, B_6_, and folate associated negatively with DMRs located near *AS3MT and GSDMD*, and positively with DMRs related to *SLC25A46* and *ZNF175* (*p *= 0.02; *p *= 0.004; *p *= 0.01; *p *= 0.006, respectively). No associations were found with individual CpGs or global DNA methylation [32].

##### Supplement use

An intervention study (Erasmus AGE score 6) randomized women to daily use of small quantity lipid-based micronutrient (SQLNS) starting at least three months before conception until delivery versus no supplements. SQLNS contain essential fatty acids, proteins, and multiple vitamins including folate, vitamin B_6_, and B_12_. Methylation of 2 CpGs in the *IGF1* promoter region were assessed. No differences were found between study groups [33].

Composition of prenatal vitamins varies but they usually contain folic acid, vitamin B_6_, and B_12_ as well as other micronutrients. *Lecorguille et al*. (ErasmusAGE score 7) found higher methylation of Alu repetitive elements in women taking vitamin supplements before and during pregnancy compared to women not taking any vitamins (β = 0.40, *p *= 0.005) although no differences in LINE-1 methylation were found. Vitamin use only before or during pregnancy was not associated with Alu or LINE-1 methylation [32].

Two studies investigated placental DNA methylation and self-reported maternal vitamin intake in the first month of pregnancy. *Dou et al*. (ErasmusAGE score 8) studied DNA methylation in the MARBLES and in the EARLI cohort, both comprise couples at increased risk for offspring with autism spectrum disorder (ASD). Mean array methylation for MARBLES women taking vitamins was −0.60% (CI −1.08, − 0.13) and the same magnitude of effect was seen in the EARLI cohort, although not significant −0.52% (CI −1.04, 0.01). No individual CpGs associated with vitamin intake using the FDR-corrected significance threshold (*p *< 1.0^−7^). With a less stringent significance threshold of *p *< 0.01, they found 9216 and 3442 differentially methylated CpGs in the MARBLES and EARLI cohort, respectively. About 95% of these showed lower methylation in the vitamin group in both cohorts (average −4%). The identified CpGs were enriched in neuronal developmental pathways [31]. *Schmidt et al*. (ErasmusAGE score 6) found no associations between vitamin use and methylation of predefined partially- or highly methylated domains or methylation of enhancers, active promoters, and bivalent promoters [38].

#### Interim conclusions – combinations

Global methylation was not associated with dietary patterns reflecting differences in intake of one-carbon moieties and use of vitamin supplements showed both increased and decreased global methylation as well as no differences depending on used technique and/or timing of supplement use. A genome-wide study revealed a few DMRs associated with dietary patterns, but dietary patterns or vitamin supplement use did not associate with individual CpGs after FDR correction. *IGF1* promoter methylation was not altered in women taking SQLNS (Table 2). In general, dietary patterns and vitamin supplement use did not substantially impact placental DNA methylation in included studies.

## Discussion

This review systematically summarizes associations between the one-carbon metabolism and placental DNA methylation. Included studies suggest that one-carbon moieties can impact placental DNA methylation at multiple specific genomic regions rather than affecting global methylation. On the other hand, this review shows that there is a lot of heterogeneity among studies regarding investigated exposures, studied outcomes, sample size, and study design. Additionally, differences in DNA methylation have been found between female and male offspring [35], depending on which part of the placenta was sampled [52], timing of exposure [32,37], and underlying genotype [42], which could also explain parts of observed differences between studies. Based on the use of ErasmusAGE quality score, we discussed studies with a quality score of at least 5 out of 10 points.

### Global Methylation

A wide variety of techniques to study global methylation have been used by included studies (Table 1). Global DNA methylation was not altered by the use of vitamin B12 supplements, while higher intake of choline did increase global DNA methylation. One study found associations between one-carbon moieties and average DNA methylation to differ between genomic regions (i.e., CpG islands, ‘shores,’ ‘shelves’ and ‘open sea’) [42]. Overall, most studies found no associations between one-carbon moieties and measures of global DNA methylation (Table 2). Hence, higher availability of methyl donors provided by the one-carbon metabolism does not just lead to increased global placental methylation.

### Genome-wide approach

The number of included genome-wide studies is limited, and only three had a quality score of at least five. All three studies used the Illumina Infinium HumanMethylation450 (450K) BeadChip array, which covers >480,000 CpGs and one partly used the Infinium methylation EPIC array covering >850,000 CpGs. All three studies investigated different exposures (Table 2). CpGs associated with maternal vitamin use in the first month of pregnancy could only be identified when a less stringent FDR-corrected p-value was applied and 95% of these were hypomethylated in women who did take vitamins in the first month of pregnancy. Identified CpGs were enriched in neuronal developmental pathways [31]. A few DMRs linked to different genes could be identified based on dietary patterns reflecting differences in intake of one-carbon moieties [32] and methylation of a few CpGs associated with umbilical cord levels of SAM, SAH, and SAM:SAH, but not with RBC folate or vitamin B_12_ levels in cord blood [42].

### Gene-specific approach

Included studies mostly investigated different loci, hampering comparison of results. Overall, most gene-specific studies did find relationships between one-carbon moieties and placental DNA methylation. On the other hand, most studies reported both significant and non-significant associations and there is a lack of uniformity in directionality of identified associations, indicating site-specific relationships (Table 2).

#### Genes involved in growth and metabolism

Genes involved in growth and metabolism have been of particular interest in included studies.

The imprinted *IGF2*/*H19* cluster plays an important role in foetal and placental growth [53]. Methylation of *IGF2/H19* has been investigated in multiple studies, with most reporting no significant associations with investigated one-carbon moieties [36,37]. In intervention studies, higher intake of choline did also not alter *IGF2* methylation [34] and the use of SQLNS did not affect methylation of *IGF1*, another major growth factor [33]. This is in contrast with other studies showing that maternal intake of methyl-group donors including folate/folic acid associated negatively with *IGF2* methylation in buccal epithelial cells in 6 months old children and positively with *IGF2* methylation in infant blood and cord blood suggesting a tissue-specific response [15,54,55].

*LEP* encodes the hormone Leptin which plays a central role in energy homoeostasis including appetite regulation and mutations in *LEP* can contribute to the development of obesity and diabetes type 2 [56]. Different results for *LEP* methylation have been reported depending on studied one-carbon moiety: positive associations with serum folate, negative associations with serum vitamin B_12_ [44], and no associations with choline have been reported [37] (Both ErasmusAGE score 6).

Cortisol regulating genes could play a role in multiple diseases including cardiovascular complications. *Jiang et al*. investigated choline intake (480mg/daily versus 930 mg/daily) and found increased methylation in the higher dose group of the *CRH* promotor and the 5’untranslated exon 1F of *NR3C1* [4]. In contrast, *NR3C1* methylation associated negatively with choline levels in a study by *Nakanishi et al*. (ErasmusAge score 6) [37]. A possible explanation is that both studies targeted a different genomic region of *NR3C1* or there may be lack of comparability between blood levels of choline and use of choline supplements.

*Nakanishi et al*. investigated several other gene-specific sites related to growth and metabolism and choline in maternal blood and cord blood. Negative associations with choline levels were also found for *AdipoQ*, *PPARG1A*, *HSD11β2*, *PPARA*, *NDUFB6*, and *RXA*, while no associations were found with methylation of *MEG3* and *MEST*, all involved in foetal growth. Differences have been found for different time points during pregnancy [7]. In an *in vitro* study, *MEST* methylation was also not altered in response to folic acid [51].

*MMP-9* codes for a matrix metalloproteinase induced during labour and MMP-9 is also increased in multiple cardiovascular diseases including hypertension and myocardial infarction [57]. Serum vitamin B_12_ and folate associated positively with *MMP-9* promoter methylation and with lower MMP-9 RNA and protein levels [41]. Contrary, folic acid treatment did not change *MMP-9* methylation in an *in vitro* model [43].

#### Genes related to placental development and function

The one-carbon metabolism has also been linked to placental angiogenesis which is crucial for a well-functioning placenta. The placenta itself produces several angiogenic factors involved in endothelial growth and function, including vascular endothelial growth factor (VEGF) and placental growth factor (PlGF), both of which are decreased in preeclampsia (PE) [58,59]. For example, increased maternal choline intake can suppress anti-angiogenic soluble fms-like tyrosine kinase 1 (*sFLT-1)* expression in human placentas and promotes angiogenesis in mice placentas [10]. Vitamin B_12_ supplements did not alter *VEGF* methylation in a high-quality intervention study [30], while another study showed *PlGF* methylation associated with SAM and SAM:SAH in the placenta [39]. Since a higher SAM:SAH ratio indicates higher availability of methyl group donors (Figure 1), a deranged one-carbon metabolism may be involved in the pathogenesis of placental-related complications partly through inducing epigenetic changes resulting in impaired placental functioning.

#### Genes directly related to one-carbon metabolism

Plasma vitamin B_12_ was negatively associated with *MTR* promoter methylation. This can have an impact on the one-carbon metabolism, since *MTR* codes for methionine synthase which is crucial in the conversion of homocysteine to methionine [40] (Figure 1).

#### Genes selected based on prior genome-wide analysis

Lastly, *Liu et al*. selected gene-specific sites of interest based on DMRs identified through genome-wide analysis. Methylation of *FAM198A* associated negatively with preconceptional start of folic acid only in male offspring indicating a sex-specific impact on DNA methylation. No associations were found between timing of folic acid supplements and methylation of 2 *ANKRD20B* CpGs [35].

In summary, most studies reported both significant and non-significant associations depending on the studied genes and moieties, indicating a site-specific relationship between the one-carbon metabolism and DNA methylation at various genes in the placenta (Table 2).

### Previous Reviews

Our review is largely in line with previously published human and animal reviews. *James et al*. focused predominantly on DNA methylation in cord blood and showed several associations between maternal one-carbon metabolism and methylation of specific genes. Overlapping genes with the current review include *IGF2/H19*, *LEP*, *NR3C1*, *RXRA*, *MEG3*, *MEST*, *IL10*, and *GNAS-AS1* and several other associations were reported. For most genes, differences were found depending on studied tissue, moiety, specific loci, and timing of exposure [12]. In rodents, maternal folic acid supplements [24] and choline intake or methyl group supplemented diets [14] have been shown to impact DNA methylation at several gene-specific sites, leading to both hypo-and hypermethylation depending on studied genomic region. Additionally, these studies found mixed outcomes for global methylation in multiple offspring tissues including brain, mucosa, and liver. For homocysteine, associations with hypomethylation were more prominent in animal models as compared to human studies, where mixed results have been reported [25]. Likewise, multiple studies using livestock showed that dietary restrictions or excesses, or a methyl supplemented diet can affect both gene-specific and global DNA methylation in the offspring, depending on studied tissue, offspring sex, and timing of exposure [26,60]. A systematic review focusing on physical activity and dietary intake of carbohydrates, fats, and proteins during pregnancy showed similar mixed associations with DNA methylation in placental tissues and cord blood [61]. This could indicate a role for a variety of lifestyle behaviours affecting the epigenome, however, the one-carbon metabolism is influenced by lifestyle behaviours which could also be (part of) the underlying biological mechanism for observed associations.

### Strengths and Limitations

In the present article, we systematically review studies investigating relationships between different one-carbon moieties and DNA methylation focusing on the human placenta. We included a wide range of exposures and outcomes and were not limited to a specific timing in relation to delivery, to be able to give a comprehensive overview of current literature. Consequently, heterogeneity between studies limits comparability of results and a meta-analysis is therefore not possible. Besides, most included studies face several challenges which should be taken into account. First, dietary patterns and vitamin use are often associated with other (lifestyle) behaviours potentially affecting DNA methylation, including smoking, BMI, and socio-economic status. Most studies are observational and do not adequately adjust for potential confounders. Second, one-carbon moieties are usually not consumed independently of each other and of other nutrients in the diet, creating difficulties when studying the effect of a single moiety. Additionally, most studies were performed in healthy cohorts without major nutritional deficiencies. The effect of differences in one-carbon moieties could be limited within a normal range. Sample size of most included studies is relatively small and self-reported exposures like food questionnaires and vitamin intake could be less reliable. Differences in sampling methods and storage conditions of placental tissues could also lead to differences between studies. Lastly, the timing between exposure and measuring outcome may affect results. Placental DNA methylation is only studied postpartum in all included studies, and might not accurately reflect methylation profiles at time of earlier exposures because of accumulating exposures during gestation also impacting DNA methylation profiles [62].

### Clinical Implications and Future Research

Current studies investigating one-carbon metabolism and placental DNA methylation display low overlap in terms of studied exposures and genomic regions, and the number of high-quality intervention studies is limited. Therefore, high-quality intervention studies in combination with a genome-wide readout for DNA methylation and preferably randomized and blinded, are warranted to further investigate the impact of an optimized maternal one-carbon metabolism on DNA methylation profiles and health in the offspring. Ideally, studies will include follow-up after birth to assess the impacts on lifelong health.

Furthermore, DNA methylation is highly cell type specific. Investigating placental DNA methylation per cell type could therefore decrease heterogeneity both within and between studies. Moreover, placental tissue might not be an appropriate model for some research questions and other tissues or cell types could also be incorporated [63]; however, tissues from internal organs are largely inaccessible and the placenta could provide insight in both placental function and foetal epigenetic programming. Generally, placental DNA methylation is only studied at one time point, i.e., after delivery. In the future, the presence of cfDNA originated from placental cell types derived from maternal blood might be an unique in vivo opportunity to non-invasively study placental DNA methylation profiles already during pregnancy [23]. Moreover, cfDNA could potentially provide targets to improve future prenatal diagnosis and obstetrical care by identifying modifiable epigenetic derangements during pregnancy, contributing to improved health outcomes across the life course.

## Conclusions

Most included studies indicate relationships between several measures of one-carbon moieties in the prenatal period and site-specific DNA methylation in the placenta rather than changes in global DNA methylation. Several gene-specific associations with DNA methylation were found, especially in genes involved in growth and metabolism. A deranged one-carbon metabolism leading to detrimental changes in the foetal epigenome in genes involved in for example the cardiovascular system may support the DOHaD paradigm. Changes in DNA methylation could not only lead to altered placental functions but could also serve as a proxy for foetal epigenetic programming, with both consequences for foetal development as well as future health outcomes.

On the other hand, conflicting outcomes were reported and there is a lack of uniformity between studies in geographically study populations, investigated loci, exposure measures, and study designs. Moreover, DNA methylation is only studied in postpartum placentas, though this may be overcome by investigating methylation of cfDNA in future studies. High-quality intervention studies tackling common limitations of included studies are needed to elucidate the relationship between one-carbon metabolism and specific differences in epigenetic programming in the offspring, ideally with longitudinal follow-up after birth. This could enable us to assess the corresponding impact on health outcomes across the life course and identify preventative or therapeutic measures contributing to improved health outcomes for future generations.

## Supplementary Material

-)Supplement Review 1CM placenta DNAm.docx

## Data Availability

The authors hereby confirm that the data supporting the findings of this systematic review are available within the article.

## List of abbreviations

ABAT: 4-aminobutyrate aminotransferase
AdipoQ: Adiponectin, C1Q and collagen domain containing
AGA: Appropriate growth for gestational age
ANKRD20B: Ankyrin repeat domain 20 family member A8, pseudogene
APC: APC regulator of WNT signaling pathway
AS3MT: Arsenite methyltransferase
ASD: Autism spectrum disorder
cfDNA: Cell free DNA
CpG: Cytosine-phosphate-Guanine
CRH: Corticotropin releasing hormone
DLL1: Delta like canonical Notch ligand 1
DMR: Differentially methylated region
DOHaD: Developmental origins of health and disease
DNMTs: DNA Methyltransferases
ETS2: ETS proto-oncogene 2, transcription factor
FAM198A: Family with sequence similarity 198 member A
FAR1: Fatty acyl-CoA reductase 1
FDR: False Discovery Rate
FFQ: Food Frequency Questionnaires
GNAS-AS1: GNAS antisense RNA 1
GNL1: G protein nucleolar 1 (putative)
GSDMD: Gasdermin D
H19: H19 imprinted maternally expressed transcript
HSD11β2: Hydroxysteroid 11-beta dehydrogenase 2
ICR: Imprinted control region
IGF1: Insulin like growth factor 1
IGF2: Insulin like growth factor 2
IL10: Interleukin 10
LEP: Leptin
LINE-1: Long interspersed nuclear elements 1
LOXL3: Lysyl oxidase like 3
MEG3: Maternally expressed 3
MEST: Mesoderm specific transcript
MMP9: Matrix metallopeptidase 9
MTHFR: Methylenetetrahydrofolate reductase
MTR: 5-methyltetrahydrofolate-homocysteine methyltransferase
NDUFB6 NADH: ubiquinone oxidoreductase subunit B6
NPDC1: Neural proliferation, differentiation and control 1
NR3C1: Nuclear receptor subfamily 3 group C member 1
P16 or CDKN2Aink4a: cyclin-dependent kinase inhibitor 2A
PE: Preeclampsia
PlGF: Placental growth factor
PPARA: Peroxisome proliferator activated receptor alpha
PEG10: Paternally expressed 10
PPARG1A: Peroxisome proliferator activated receptor gamma
RAD54L2: RAD54 like 2
RBC: Red blood cell
RXRA: Retinoid X receptor alpha
SAH: S-adenosylhomocysteine
SAM: S-adenosylmethionine
SGA: Small for gestational age
SLC25A46: Solute carrier family 25 member 46
SNRPN: Small nuclear ribonucleoprotein polypeptide N
SQLNS: Small quantity lipid-based micronutrient
VEGF: Vascular endothelial growth factor
ZC3H12D: Zinc finger CCCH-type containing 12D
ZNF175: Zinc finger protein 175
ZNF836: Zinc finger protein 836
