# Supplementary material for: The one-carbon metabolism as an underlying pathway for placental DNA methylation – a systematic review
Source: Epigenetics. 2024 Mar 14;19(1):2318516. doi: 10.1080/15592294.2024.2318516 (PMC10950272; doi:10.1080/15592294.2024.2318516)
Supplement: -)Supplement Review 1CM placenta DNAm.docx [file KEPI_A_2318516_SM6786.docx]

Supplementary material

**Table S1**: **Used search strategies**

| **Database** | **Searching strategies** | **Results (n)** |
| --- | --- | --- |
| **Medline** | (exp Vitamins/ OR exp Avitaminosis/ OR Homocysteine/ OR Life Style/ OR Diet/ OR "Diet, Food, and Nutrition"/ OR Nutritional Sciences/ OR Malnutrition/ OR Feeding Behavior/ OR Diet, Healthy/ OR exp Dietary Supplements / OR Antioxidants/ OR Metabolome/ OR Choline/ OR Maternal Nutritional Physiological Phenomena/ OR Oxidants/ OR Nutritional Status/ OR "Methylenetetrahydrofolate Reductase (NADPH2)"/ OR ((carbon ADJ3 metabol*) OR one-carbon OR 1-carbon OR vitamin* OR avitaminos* OR folic-acid* OR folate* OR homocystein* OR life-style* OR lifestyle* OR diet OR nutrition* OR malnutrition* OR nutrient* OR micronutrient* OR supplement* OR antioxidant* OR anti-oxidant* OR methylenetetrahydrofol* OR mthfr OR metabolom* OR cholin* OR ((parent* OR maternal* OR paternal* OR mother* OR father*) ADJ3 (intake* OR deficien* OR status)) OR (zinc ADJ3 (status* OR metabol* OR intake* OR deficien*)) OR (dietar* ADJ3 ratio*) OR cyanocobalamin* OR mecobalamin* OR methylcobalamin* OR cobalamin* OR cobamamid* OR hydroxocobalamin*).ab,ti.) AND (exp Placenta / OR exp Placenta Diseases / OR (placenta* OR Uteroplacenta*).ab,ti.) AND (DNA Methylation / OR Epigenomics / OR Epigenome / OR Histone Code / OR DNA Damage / OR Epigenesis, Genetic / OR (((DNA OR cfDNA OR cffDNA OR histone OR gene) ADJ6 (methylat* OR hypermethylat* OR hypomethylat* OR modificat* OR damage)) OR epigenetic* OR epigenom* OR methylome* OR (genetic* ADJ3 epigenes*)).ab,ti.) AND english.la. | 699 |
| **Embase** | ('carbon metabolism'/de OR vitamin/exp OR homocysteine/de OR lifestyle/exp OR diet/de OR nutrition/de OR 'feeding behavior'/de OR 'healthy diet'/de OR 'unhealthy diet'/de OR supplementation/de OR 'vitamin supplementation'/de OR 'mineral supplementation'/de OR antioxidant/de OR metabolome/de OR choline/de OR 'maternal nutrition'/de OR 'nutritional deficiency'/exp OR 'oxidizing agent'/de OR 'diet supplementation'/de OR 'nutritional status'/de OR 'methylenetetrahydrofolate reductase (NADPH2)'/de OR 'zinc metabolism'/de OR 'zinc deficiency'/de OR 'zinc intake'/de OR ((carbon NEAR/3 metabol*) OR one-carbon OR 1-carbon OR vitamin* OR avitaminos* OR folic-acid* OR folate* OR homocystein* OR life-style* OR lifestyle* OR diet OR nutrition* OR malnutrition* OR nutrient* OR micronutrient* OR supplement* OR antioxidant* OR anti-oxidant* OR methylenetetrahydrofol* OR mthfr OR metabolom* OR cholin* OR ((parent* OR maternal* OR paternal* OR mother* OR father*) NEAR/3 (intake* OR deficien* OR status)) OR (zinc NEAR/3 (status* OR metabol* OR intake* OR deficien*)) OR (dietar* NEAR/3 ratio*) OR cyanocobalamin* OR mecobalamin* OR methylcobalamin* OR cobalamin* OR cobamamid* OR hydroxocobalamin*):Ab,ti) AND (placenta/exp OR 'placenta tissue'/de OR 'placenta function'/exp OR 'placenta disorder'/exp OR (placenta* OR Uteroplacenta*):Ab,ti) AND ('DNA methylation'/exp OR epigenetics/exp OR epigenome/exp OR 'histone modification'/exp OR 'DNA damage'/exp OR 'genetic epigenesis'/de OR (((DNA OR cfDNA OR cffDNA OR histone OR gene) NEAR/6 (methylat* OR hypermethylat* OR hypomethylat* OR modificat* OR damage)) OR epigenetic* OR epigenom* OR methylome* OR (genetic* NEAR/3 epigenes*)):Ab,ti) NOT ([conference abstract]/lim AND [2000-2019]/py) AND [english]/lim | 1046 |
| **Web of Science** | TS=((((carbon NEAR/2 metabol*) OR one-carbon OR 1-carbon OR vitamin* OR avitaminos* OR folic-acid* OR folate* OR homocystein* OR life-style* OR lifestyle* OR diet OR nutrition* OR malnutrition* OR nutrient* OR micronutrient* OR supplement* OR antioxidant* OR anti-oxidant* OR methylenetetrahydrofol* OR mthfr OR metabolom* OR cholin* OR ((parent* OR maternal* OR paternal* OR mother* OR father*) NEAR/2 (intake* OR deficien* OR status)) OR (zinc NEAR/2 (status* OR metabol* OR intake* OR deficien*)) OR (dietar* NEAR/2 ratio*) OR cyanocobalamin* OR mecobalamin* OR methylcobalamin* OR cobalamin* OR cobamamid* OR hydroxocobalamin*)) AND ((placenta* OR Uteroplacenta*)) AND ((((DNA OR cfDNA OR cffDNA OR histone OR gene) NEAR/5 (methylat* OR hypermethylat* OR hypomethylat* OR modificat* OR damage)) OR epigenetic* OR epigenom* OR methylome* OR (genetic* NEAR/2 epigenes*)))) AND DT=(article) AND LA=(english) | 639 |
| **Cochrane CENTRAL Register of Controlled Trials** | (((carbon NEAR/3 metabol*) OR one-carbon OR "1-carbon" OR vitamin* OR avitaminos* OR folic-acid* OR folate* OR homocystein* OR life-style* OR lifestyle* OR diet OR nutrition* OR malnutrition* OR nutrient* OR micronutrient* OR supplement* OR antioxidant* OR anti-oxidant* OR methylenetetrahydrofol* OR mthfr OR metabolom* OR cholin* OR ((parent* OR maternal* OR paternal* OR mother* OR father*) NEAR/3 (intake* OR deficien* OR status)) OR (zinc NEAR/3 (status* OR metabol* OR intake* OR deficien*)) OR (dietar* NEAR/3 ratio*) OR cyanocobalamin* OR mecobalamin* OR methylcobalamin* OR cobalamin* OR cobamamid* OR hydroxocobalamin*):Ab,ti) AND ((placenta* OR Uteroplacenta*):Ab,ti) AND ((((DNA OR cfDNA OR cffDNA OR histone OR gene) NEAR/6 (methylat* OR hypermethylat* OR hypomethylat* OR modificat* OR damage)) OR epigenetic* OR epigenom* OR methylome* OR (genetic* NEAR/3 epigenes*)):Ab,ti) | 27 |
| **Google Scholar** | "carbon metabolism"\|"one-carbon"\|vitamins\|"folic-acid"\|folate\|  homocystein\|"life-style"\|lifestyle\|diet\|nutrition\|nutrients\|supplements placenta "DNA\|histone\|gene methylation\|hypermethylation\|  hypomethylation\|modification\|damage"\|epigenetics\|epigenomics | 200 |

**Table S2: ErasmusAGE quality score form for systematic reviews**

Original: ErasmusAGE, 24 June 2013

This quality score can be used to assess the quality of studies included in systematic reviews and meta-analyses and is applicable to both interventional and observational studies. The score was designed based on previously published scoring systems (Carter et al, 2010 and the Quality Assessment Tool for Quantitative Studies). The quality score is composed of 5 items, and each item is allocated 0, 1 or 2 points. This allows a total score between 0 and 10 points, 10 representing the highest quality. The version presented below needs to be adapted for each review separately.

1. **Study design**

**0** for studies with cross-sectional data collection

**1** for studies with longitudinal data collection (both retrospective and prospective)

**2** for intervention studies

1. **Study size** (predefined)*

**0** small population for analysis ( <50 patients)

**1** intermediate population for analysis (50-150 patients)

**2** large population for analysis (>150 patients)

1. **Exposure**

*Observational studies*

**0** if the study used no appropriate exposure measurement method or if not reported

**1** if the study used moderate quality exposure measurement methods

**2** if the study used adequate exposure measurement methods

*Intervention studies*

**0** if the intervention was not described or not blinded

**1** if the intervention was adequately single blinded.

**2** if the intervention was adequately double-blinded.

1. **Outcome**

**0** if the study used no appropriate outcome measurement method or if not reported

**1** if the study used moderate quality outcome measurement methods

**2** if the study used adequate outcome measurement methods

1. **Adjustments** †*

**0** if findings are not controlled for at least key confounders (fetal sex, gestational age)

**1** if findings are controlled for key confounders (fetal sex, gestational age)

**2** if findings are additionally controlled for additional covariates or when an intervention is adequately randomized
